# Usefulness of Patients-Reported Outcomes in Rheumatoid Arthritis Focus Group

**DOI:** 10.1155/2012/935187

**Published:** 2012-09-28

**Authors:** Jenny Amaya-Amaya, Diana Botello-Corzo, Omar-Javier Calixto, Rolando Calderón-Rojas, Aura-Maria Domínguez, Paola Cruz-Tapias, Gladis Montoya-Ortiz, Ruben-Dario Mantilla, Juan-Manuel Anaya, Adriana Rojas-Villarraga

**Affiliations:** ^1^Center for Autoimmune Diseases Research (CREA), School of Medicine and Health Sciences, Universidad del Rosario, Bogota, Colombia; ^2^Riesgo de Fractura-Cayre IPS, Rheumatology Unit, Bogota, Colombia; ^3^Doctoral Program in Biomedical Sciences, Universidad del Rosario, Bogota, Colombia

## Abstract

*Objective*. Patient-reported outcomes (PROs) have become an essential part of the assessment of patients with rheumatoid arthritis (RA). We aimed to evaluate the agreement and correlation between PROs and the physician's measurements. *Methods*. This was a cross-sectional analytical study in which 135 patients with RA were clinically evaluated during two different sessions of focus group interviews. Rheumatologist recorded 28 swollen (SJCs) and tender joint counts (TJCs). The patients filled out the PROs instruments (MDHAQ, RADAI, RAPID3, 4, and 5 and self-report articular index (SAI) diagram for pain and joint swelling). DAS28 was calculated (C-reactive protein). An adjusted multiple lineal regression model was done (DAS28 as dependent variable). *Results*. Highly significant agreements were found between SJC and TJC registered by the physician and patient. There was moderate correlation between DAS28 with patient SJC (*r* = 0.52), patient TJC (*r* = 0.55), RADAI (*r* = 0.56), RAPID3 (*r* = 0.52), RAPID4 (*r* = 0.56), RAPID5 (*r* = 0.66), and VAS-Global (*r* = 0.51). Likewise, we found moderate to high correlations between CDAI and SDAI with all variable measurements done by the patients. The resulting predictive equation was DAS28(CRP) = 2.02 + 0.037 × RAPID4 + 0.042× patient SJC. *Conclusion*. PROs applied in focus groups interview are a useful tool for managing patients with RA regardless of gender, educational level, and duration of disease.

## 1. Introduction

Rheumatoid arthritis (RA) is a chronic, complex, heterogeneous, and widely known autoimmune disease (AD). It is characterized by the presence of long-standing inflammation of the diarthrodial joints resulting in symmetric polyarthritis and synovial membrane hypertrophy with progressive damage to the joints, bone and cartilage destruction, and deformity. However, the autoimmune compromise is systemic and thus, leads to extra articular manifestations (EAMs) including cutaneous nodules, lung involvement, cardiovascular disease (CVD), episcleritis, and vasculitis [1–3]. All of these lead to an increase in comorbidities [4, 5], disability [6, 7], impaired quality of life [8, 9], and premature mortality, which is two times the general population [10, 11]. 

The disease is more frequent in women than men [5, 12, 13]. The age at onset is commonly situated around the 30s with a peak in the fifth decade of life according to the majority of epidemiological studies [14]. Several incidences and prevalence of the disease have been reported during the last few decades which suggest a high admixture of cultures, ethnics, environmental, genetic, and epigenetic factors. The majority of studies carried out in Northern Europe and North America estimate a prevalence of 0.5-1.1% [12, 15]. Studies from developing countries report lower prevalence (between 0.1–0.5%) even in Latin America population [12, 16]. The worldwide incidence rates (cases per 100 inhabitants) oscillate from 0.01 in Southern Europe to 0.3 in Asia [12]. Furthermore, the incidence increases with age and seems to reach a plateau as of the age of 60 [13]. Incidence in the United States, in turn, is estimated to be 25 per 100,000 persons for men and 54 per 100,000 persons for women [3].

Considering that RA is the most common inflammatory arthropathy worldwide and causes multiple disabilities, an inadequate assessment of clinical status can lead to inappropriate treatment and undesirable outcomes. It is necessary to implement clinical measures to determine the degree of activity and disease involvement. Traditionally, evaluation of RA has centered around physician-generated assessments in clinical outpatient care with many restrictions such as a limited amount of time in consultation, absence of a gold standard for diagnosis and subsequent followup [17, 18], and the lack of patient participation [19–21]. 

Currently, the evaluation of a RA patient involves aspects of the disease pathophysiology (i.e., measurement of C-reactive protein (CRP), erythrocyte sedimentation rate (ESR), lipid profile, antibodies, and X-ray), disease activity, functional capacity, structural damage, pain, fatigue, and quality of life. All these allow a better and more objective assessment, which includes the most relevant long-term outcomes [22], presence or absence of comorbidity, drug toxicity, psychological and social consequences, prognosis, premature mortality, and high disease costs [6, 19, 23–25]. 

In recent years, there has been a growing interest in the assessment of patients with RA from the patient's perspective. Patient-reported outcomes (PROs) in RA are processes in which the patient completes some forms (i.e., questionnaire, scales, self-administered index (SAI) diagram) and objectively evaluates the disease. It has been found to be as or more informative than physician-assessed measurement because it allows the information necessary for clinical and therapeutic decisions to be collected. The information is organized into quantitative data and used to make decisions as well as assess the prognosis and most probable outcomes for the patients [24, 26–30].

In both clinical practice and research, the PROs, though they are self-report tools, have been designed, validated, reliable and reproducible world-wide [31–34]. Most studies have been able to demonstrate agreement between self-administered and observed-derived assessment of joint counts, and so forth [17, 30, 35–39]. This agreement allows these qualitative data to be summarized and converted into quantitative data classified by scores. This makes an objective and reproducible assessment that can be used over time possible during the visits to the rheumatologist. 

In order to demonstrate the agreement and correlation present between PROs and the measurements from the physician in RA patients, a cross-sectional study was done to evaluate the agreement and usefulness of PROs in comparison to objective measurements during a focus group of Colombian RA patients. 

## 2. Material and Methods

### 2.1. Study Population

This was a cross-sectional analytical study in which 135 consecutive patients with RA were included. All of them fulfilled the 1987 American College of Rheumatology classification criteria [40] and were seen at three different outpatient clinics in Bogota, Colombia. Also, they were contacted by telephone, brought together, and clinically evaluated during two different sessions of focus group interviews. Each session included approximately 70 patients. This study was undertaken between November 2010 and January 2011 and done in compliance with Act 008430/1993 issued by the Ministry of Health of the Republic of Colombia. The ethics committee of the Universidad del Rosario approved the study design. 

The focus groups interview methodology was coordinated by a rheumatologist who explained the concept of PROs, the activities, and the tools used for gathering the information (i.e., questionnaires and SAI diagram [38]). After that the patients filled out the questionnaires with information about sociodemographic and cumulative clinical data. Most patients were able to complete the instruments with no problem. However, if requested by the patient, ten health care providers helped them complete the questionnaires. After the focus group interview, physicians through chart, radiographic review, and telephone interview confirmed the data collected. 

The questionnaires used by the patients for the self-report were

**Figure 1:**
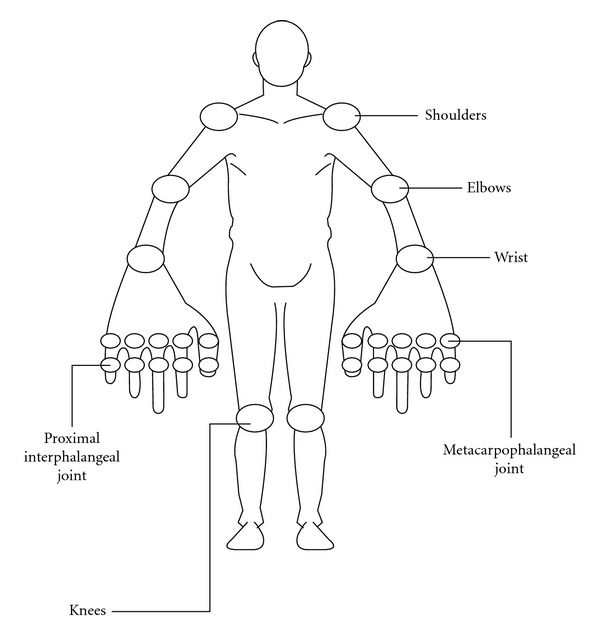
Self-administered index (SAI) Modified from [38].

- multidimensional health assessment questionnaire R729-NP2 (MDHAQ), Spanish version [41];
- pain visual scale analogue (VAS-Pain) (0–10);
- self-administered, rheumatoid arthritis disease activity index (RADAI), where the patient self-reported tender joints on a scale of 0–3 from 8 bilateral joint groups (0–10) [30];
- global assessment by visual scale analogue (VAS-Global) (0–10);
- swollen joint count (SJC) and tender joint count (TJC) in the SAI [38], (Figure 1).

Each patient was examined by a rheumatologist who determined:

- out of a total of 28 joints the physician identified and TJC by physical examination. This examination was blinded and done independently of the questionnaires filled out by the patients;
- global assessment by visual scale analogue (MD-Global) (0–10);
- anthropometric measurements;
- after the informed consent was signed, a blood sample was drawn for the CRP measurement.

These composite indices were determined in each patient:

- RAPID3: (routine assessment of patient index data) [42]. This is a PROs-based index that uses the three core set criteria evaluated by the patient, that is, physical function (from MDHAQ), VAS-Pain, and VAS-Global (scale 0–10);
- RAPID4: [43] this includes the same variables as RAPID3 plus RADAI (Scale 0–10);
- RAPID5: [43] this includes the same variables as RAPID4 plus MD-Global (Scale 0–10);
- DAS28-CRP: (disease activity score-28 joints) [44]. It is made up of the TJC and SJC on 28 joints determined by physician and CRP (mg/L). The equation is as follows: DAS28=0.56∗(TJC28)+0.28∗(SJC28)+0.36∗ln⁡⁡(CRP+1)∗1.10+1.15;
- SDAI: simplified disease activity index [45] is the algebraic sum of the following five parameters: TJC and SJC on 28 joints determined by the physician, CRP level in mg/dL, patient VAS-Global, and MD-Global;
- CDAI: clinical disease activity index [46] is the algebraic sum of the SDAI items minus the CRP level;
- conversion from MDHAQ to the original health assessment questionnaire (HAQ) though Anderson's model [47].

The sociodemographic variables included current age, age at RA onset, disease duration, educational status, socioeconomic status (SES), current occupational status, smoking habits, coffee consumption, and physical activity. The following are the definitions of these variables (Table 1): age at onset is age at which patients began to suffer from pain, typical morning stiffness (more than 1 hour), and symmetrical inflammation of hand and/or foot joints. Disease duration is difference between age at onset and the date of first participation in the study. It was divided into either more or less than 10 years of disease as our group had previously reported this to be a risk factor for poor prognosis (i.e., CVD) [48]. Educational level was recorded as years of education. These data were dichotomized into two groups with one group including those with less than 9 years of education (including preschool, primary, and the first 2-3 years of high school) and the other group more than 9 years of education. This breakdown was based on the General Law of Education in Colombia [49, 50]. SES was categorized on the basis of national legislation and was divided into high status (3 to 6) and low status (1 and 2). For occupational status, we focused on establishing if the patient worked at household duties exclusively.

Regarding clinical variables, polyautoimmunity, multiple autoimmune syndrome (MAS), familial autoimmunity, erosions, comorbidities, EAMs, systolic and diastolic blood pressure, body mass index (BMI), and waist circumference were evaluated. The following are the definitions of these variables. Polyautoimmunity is the presence of more than one autoimmune disease in a single patient [51]. MAS corresponds to the coexistence of three or more well-defined ADs [51]. In order to define these two, we evaluated 6 ADs on the basis of international criteria, that is, systemic lupus erythematosus (SLE) [52], autoimmune thyroid disease (AITD), Sjögren's syndrome (SS) [53], antiphospholipid syndrome (APS) [54], scleroderma (SSc) [55], and vitiligo [56]. Familial autoimmunity was defined as the presence of any diagnosed AD in any first-degree relatives (FDR) of the proband [57]. AITD was confirmed on the basis of an abnormal thyrotropin (TSH) test or history of thyroid hormone therapy and the presence of either antibodies, antithyroperoxidase enzyme (TPOAb), or antithyroglobulin protein (TgAb). 

Erosions were defined as having at least one unequivocal cortical bone defect evaluated by two blinded researchers (a rheumatologist and a radiologist) [58]. EAMs was defined as the presence of at least one of the following: skin ulcerations, nodules, episcleritis, vasculitis, neuropathy, pleural effusion, pulmonary hypertension or embolism, and CVD. The latter was categorized as positive if any of the following variables were present: hypertension (defined as having a blood pressure >140/90 mm Hg or using antihypertensive medication) [59], coronary artery disease, occlusive arterial disease, carotid disease, or thrombosis [60].

The patients were asked about the presence of diabetes mellitus, defined as having a fasting plasma glucose level > 7 mmol/L (126 mg/dL) or taking antidiabetic medication at the time of the assessment [61]. Diagnosis of dyslipidemia was given if the patient had hypercholesterolemia, defined as taking lipid-lowering medication or having a fasting plasma total cholesterol >200 mg/dL, HDL < 40 mg/dL, hypertriglyceridemia > 150 mg/dL, or LDL cholesterol > 100 mg/dL [62]. Anemia was diagnosed if current hemoglobin was <12 g/dL, gastritis only if evidenced by esophagogastroduodenoscopy, periodontal disease was self-reported, and renal disease if the serum creatinine measurement had values above 1.2 mg/dL.

Systolic and diastolic blood pressures were measured twice with at least 15 minutes between measurements and the averages were recorded. A BMI ≥ 25 kg/m^2^ (overweight and obesity) was considered abnormal [63]. Abnormal values of waist circumference (>102 cm for men, >88 cm for women) and waist-to-hip ratio (WHR; >0.9 for men, >0.85 for women) were considered indicators of abdominal obesity. Waist circumference was measured around the narrowest point between ribs and hips after exhaling and viewed from the front. Hip circumference was measured at the point of maximum extension of the buttocks when viewed from the side [64]. Abnormal WHR values are consistent with National Cholesterol Education Program Adult Treatment Panel III and World Health Organization definitions [65]. 

Medical treatment includes the current or past use of methotrexate and other disease modifying antirheumatic drugs (DMARDs) such as sulfasalazine, D-penicillamine, azathioprine, cyclosporine, gold salts and leflunomide, steroid therapy, antimalarials (chloroquine, hydroxychloroquine), and biological therapy (rituximab, infliximab, etanercept, abatacept, adalimumab, or tocilizumab). Patients and their past medical records were evaluated for the current or past use of aspirin or hormone replacement therapy as well.

Relevant laboratory variables were also registered including ESR, hemoglobin levels, white blood cell count, platelet count, and highly sensitive CRP serum levels. Autoantibodies such as rheumatoid factor (RF) and anticyclic citrullinated peptide (anti-CCP), antinuclear antibodies (ANAs), Ro, La, RNP, Sm, IgG, and IgM anticardiolipins, and TPOAb and TgAb antibodies were taken from the patient's clinical record. They were measured with enzyme-linked immunosorbent assay (QUANTA-Lite, INOVA, San Diego, CA, USA) following the manufacturer's protocol. Antibodies directed against either TSH receptor or thyroid hormones (THAb) were not assessed in the current study.

### 2.2. Statistical Analysis

First, univariate analysis was done. Categorical variables were analyzed by frequencies. Kolmogorov-Smirnov normality test was done to evaluate normality for quantitative variables. Parametric data are expressed as mean and standard deviation (SD), and nonparametric data are described as median and interquartile range (IQR). 

Agreement and correlation between patient and rheumatologist variables were evaluated by the statistic tests described in the footnote of Table 2. We considered correlations between 0.5 and 0.7 to be moderate and correlations of more than 0.7 to be high [66, 67]. 

To assess predictors for DAS28 (objective measurement), variables that had significant correlations with DAS28 (dependent variable) were entered as independent variables in the multiple lineal regression model (multivariate analyses). Those variables were patient SJC and TJC (SAI diagram), RADAI and RAPID4. The last two were considered crude data (values between 0–48 and, 0–40 respectively). MDHAQ, VAS-Global, VAS-Pain, and RAPID3 were not included due to the fact that these are contained in RAPID4, RAPID5 was also excluded because it included MD-Global (an objective measurement). This model was adjusted by gender, duration of the disease, and educational level. The adequacy of lineal regression models was assessed using the Durbin-Watson goodness-of-fit test. Statistical analyses were done by using the Statistical Package for the Social Sciences (SPSS, v.20, Chicago, IL, USA).

## 3. Results

Table 1 describes the main sociodemographic, clinical, and autoimmune characteristics. Out of a total of 135 patients, 78.59 % were women. The most frequently reported occupation was household duties at 36.3% (49/135), and the most frequently reported comorbidity was osteoporosis at 31.1% (42/135). 

A positive RF was registered as positive in 85.5% and anti-CCP was positive in 89.2% of the cases (Table 1). A total of 64.4% of the patients had at least one EAMs with the presence of CVD and nodules being the most frequent (Table 1). Steroids and methotrexate were the most frequently used medications. Polyautoimmunity was present in 14.1% with AITD as the most frequent coexistent AD. 

According to the calculation of the RAPID3, 4, and 5, 53.3% (72/135), 51.8% (70/135), and 27.4% (37/135) respectively, had high scores, which indicated severe activity of the disease. Table 2 shows the correlation of values between the measurements done by the rheumatologist and patient. Highly significant (*P* < 0.0001) agreements were found between SJC and TJC registered by the physician and patient. There was a moderate correlation (*P* < 0.0001) between DAS28 with patient SJC (*r* = 0.52), patient TJC (*r* = 0.55), RADAI (*r* = 0.56), RAPID3 (*r* = 0.52), RAPID4 (*r* = 0.56), RAPID5 (*r* = 0.66), and VAS-Global (*r* = 0.51). Likewise, we found moderate to high correlations between CDAI and SDAI with all variable measurements done by the patients. The correlation between either CDAI or SDAI and RAPID5 was the highest (*r* = 0.82 and *r* = 0.85).

In the multiple lineal regression model (Table 3), the resulting predictive equation was DAS28(CRP) = 2.02 + 0.037 × RAPID4 + 0.042 × patient  SJC. Other independent variables were not significant in the DAS28 prediction. The educational level, duration of the disease, and gender did not have an influence on the predictive model. The explanation from the model was 40% (*R* ^2^). Correlations between the residuals (Durbin Watson = 2.26) and multicollinearity between independent variables (variance inflation factor < 10) were not found.

## 4. Discussion 

In the current study, agreement was found between objective measurements assessed by the physician and subjective assessments done by the patient, which highlight the agreement between SJC and TJC as well as the correlation between activity index (CDAI and SDAI) and all the variables measured by the patient. Even though these tools are widely known since they provide the physician with information about the disease course and red flags, they are not usually applied in the daily routine with individual patients but rather in clinical research [17, 24, 68]. We also found that RAPID4 and SJC from patients can be used to predict DAS28. Therefore, we confirmed that the PROs, administered in focus group sessions with RA patients, are an objective approach to disease [42]. 

### 4.1. General Aspects of PROs Instruments

Quantitative assessment in RA differs from the assessment of many other clinical conditions because a single gold standard measurement is not available to evaluate the complete individual disease activity of the patient. Practicing rheumatologists might have insufficient time to do a complete disease activity and functional status evaluation during every patient visit [19]. Most standard rheumatology care continues to be handled largely on the basis of laboratory tests (i.e., CRP, ESR, antibodies) and radiographic scores combined with subjective judgment without formal quantitative joint counts or patient questionnaires [68, 69]. Nonetheless, concerning functional status, patient questionnaires provide the most significant prognostic clinical measurement for all important long-term outcomes of RA including functional status, work disability, costs, joint replacement surgery, and premature death [70, 71]. However, psychological issues, depression, and anxiety, among others, are also important to evaluate through scales and questionnaires [72]. All these objective measurements assist the physician in guiding assessment, management, and prognosis for each patient, while these are filled out in the waiting room [20, 41, 73]. 

Nevertheless, objective measurements are not without some limitations. These include the time required to compute and interpret the scales. For instance, calculating the DAS28-CRP or DAS28-ESR requires a calculator, computer or web site, and the time spent is 114 seconds. Computing CDAI takes 106 seconds [18, 41, 74–76]. Furthermore, each one requires different scales and cutoff points to interpret it. In contrast, RAPID3 on an MDHAQ can be calculated in 5 to 10 seconds [41]. 

Additionally, a complete joint count, which is usually not done by a large percentage of rheumatologists, is necessary. Sometimes the fact that they do not do the joint count causes them to lose interest in the use of these measurements [38, 41, 42, 68, 77]. Another disadvantage is that the primary concerns of patients and their families are not addressed [78, 79]. 

Due to the difficulties and limitations mentioned above, PROs have been designed to guide clinical care complemented by objective measurements done by the physician. A PROs are anyreport coming directly frompatients, without interpretation by physicians or others, about how they function or feel in relation to a health condition and its therapy [80]. PROs instruments are used to measure thesepatientreports. Common examples of PROs include quality-of-life and health status measurements, patient satisfaction and experience, psychological distress, pain, and self-efficacy. The common feature of PROs measurements is their grounding in the patient's perspective. PROs assessments are typically obtained through self-administered questionnaires, self-report scales, mannequins, and so forth. in the waiting area, by telephone, via postal mail, or online. PROs have been implemented globally and have correlated significantly with objective values in rheumatologic diseases and other chronic pathologies (i.e., cancer, asthma, hypertension, heart disease, stroke, psychiatric illness, migraines, diabetes) [26, 80–84].

Standardized patient measurement tools, rather than laboratory tests, are the most significant quantitative predictors of severe outcomes in many chronic diseases [24, 75]. These PROs instruments are useful for monitoring patient status over time due to their validity, reliability, feasibility, and their sensitivity to change. All these features can improve and optimize the time in the visit to the doctor by providing additional time for a complete physical examination. Otherwise, PROs improve the physician-patient relationship [38, 85], ease implementation of educational tools, which strengthens self-assessment of doctor care, diminish feelings of disability and risk of depression, promote a return to an active role in society, and strengthen social support. Furthermore, the patients become active participants in their followup, their adherence to the treatment improves, and there is greater disease control and a better prognosis [81–83]. 

In recent years, there has been growing interest in the assessment of patients with RA from the patient's perspective. The importance of PROs has been increasingly recognized over the years, and there are several reasons for the growing popularity of assessing PROs in rheumatology. 

Patient medical history may be recorded as standardized ‘‘scientific” quantitative data on validated self-report questionnaires. Data from patient questionnaires are as effective as or more effective than laboratory tests and joint count data in discriminating active from control treatments in clinical trials and outpatient clinical care [29, 79, 86]. For instance, the most significant marker for predicting premature mortality over 5 years in patients with RA is a score for functional capacity in activities of daily living on a patient questionnaire rather than currently available laboratory tests, radiographs, or other imaging data [22, 87]. In a study of patients who had an extensive baseline evaluation in 1973 and were reviewed 9 years later in 1982, patient responses regarding capacity to carry out their usual activities predicted mortality 5 years later more effectively than any known clinical measure. Patients who could do fewer than 80% of their daily living activities ‘‘with ease” according to a questionnaire experienced a 5-year survival of about 50%, which is in the same range as patients with Stage IV Hodgkin's disease and 3-vessel coronary artery disease [88, 89]. Similar findings have been reported by Sokka et al. [90], Callahan et al. [89], and Wolfe et al. [91] with functional status measured by HAQ and MDHAQ. 

### 4.2. Grade of Agreement between Physician and Patient Measurements

RAPID3 is an index proposed for the assessment and management of patients with RA that includes only the 3 patient-reported American College of Rheumatology (ACR) Core Data Set measurements, without formal joint count, for RA: physical function, pain, and VAS-global of status. It can be calculated in 5 to 10 seconds, in contrast to the 90 to 94 seconds for a formal 28-joint count, 106 seconds for a CDAI, and 114 seconds for a DAS28 [42]. Leeb et al. [17, 73] reported a substantially lower agreement between RAPID3 and DAS28, *r* = 0.32 and RAPID3 and CDAI, *r* = 0.37. In contrast, Pincus et al. [35, 92] demonstrated Spearman rank order correlation coefficients of 0.66 for DAS28-ESR with RAPID3, 0.50 for DAS28-CRP with RAPID3, and 0.74 for CDAI with RAPID3. All of these were highly significant (*P* < 0.001). Our findings are similar with Spearman's rank correlation coefficients of 0.52 for DAS28-CRP with RAPID3 and 0.73 for CDAI with RAPID3. Both of these were highly significant (*P* < 0.001). 

Likewise, RAPID4 measures a construct of RA clinical status similar to DAS28 and CDAI because it includes RAPID3 and RADAI, a validated self-report joint count. RAPID4 can be calculated in about 19 seconds [93]. So far we have found agreement between RAPID4 and TJC, DAS28, CDAI, and SDAI, and there was no correlation with SJC. This could be due to the fact that the tender joint sub-score contributed only 17% of the total RAPID4 score [94] and that RADAI includes only painful joints. 

RAPID3, RAPID4, and RAPID5 give similar results that distinguish between active disease and that controlled by treatment in RA clinical trials just as ACR improvement criteria do. All of these correlate significantly with DAS28 [35, 43, 70, 95]. Our findings agree with the above results and the correlation coefficients were 0.52, 0.56, and 0.66 between DAS28 and RAPID3, RAPID4, and RAPID5, respectively. 

As noted, the joint count is the most specific measurement to assess RA. Several types of self-report joint counts have been reported since the 1980s showing correlation at levels of *r* = 0.44–0.87 with traditional TJC [30, 36, 96, 97]. RADAI self-report joint count correlates significantly with a physician/assessor TJC [30, 98, 99] as we demonstrated in the present study (*r* = 0.60, *P* < 0.001).

MDHAQ is a PROs instrument developed to include 6 complex activities of daily living which reflect status of patients currently seen by rheumatologists [74]. The reports of the HAQ and MDHAQ suggest that patient self-report data were generally more reliable than data elicited by a health professional observer, and these have been correlated with activity indices such as DAS28, CDAI, and SDAI [100–104] which is correlated with our findings. The greater reliability of self-reported data can be largely explained by the fact that the measurement was done only once by a single observer, the patient, rather than the two observers (i.e., the patient and a health professional) [93].

CDAI and SDAI, in turn, are measurements having a moderate to high correlation with all variables measured by the patient in the present study. For instance Rintelen et al. [105] also found a highly significant relationship between SDAI/CDAI levels and the patient's pain rating (SDAI: *r* = 0.660, *P* < 0.001; CDAI: *r* = 0.671, *P* < 0.001). SDAI was highly correlated with the patient VAS-Global (*r* = 0.72, *P* < 0.001) in our cohort just as Leeb et al. [103] had shown in 2004. 

### 4.3. Advantages and Weakness of PROs

The correlations between measurements taken by the physician and the patients show advantages in their management and prognosis of their disease. PROs had reported an association and are far more significant than laboratory tests or radiographs [24] for predicting, as mentioned above, premature mortality, costs, work disability, joint replacement, and premature death [106–110]. Other benefits of PROs in RA are the capacity to distinguish active disease from that controlled by treatment as DAS28 and CDAI do. The three also have a significant correlation with joint counts, ESR, and X-ray scores and are equally or proportionately as informative as the ACR 20, 50, 70 or DAS. Therefore, the patient may serve as his own “control” over time [20, 78]. In addition, they are more reproducible and less likely to improve with a placebo than traditional joint counts, ESR, X-ray scores, and physical measurements. It allows differentiation between case and control groups in phase III clinical trials and the modification in the treatment of placebo groups [20, 92, 111–113]. 

On basis of PROs, the physician can arrange strategies for monitoring patients at each visit based on the fact that the scores are available on a flow sheet, which allows the latest visit to be compared to previous ones before seeing the patient. Low cost and easy application are other features of these questionnaires and scales [24, 78]. Thus, physicians need little time to calculate questionnaires, (i.e., MDHAQ, RAPID) without mathematic formulas, advanced calculators, or quantitative articular count [18, 74, 76]. This has been reasonably shorter than the time necessary to calculate a DAS28 or a CDAI [17, 114, 115].

The questionnaire should be distributed to each patient at each visit. They complete the PROs instruments which are valid, reliable, effective, easily administered, and scored as a component of the infrastructure of standard rheumatology care [93]. Thus, the PROs instruments help the patient prepare for the visit by completing it in the waiting area prior to seeing the physician. The clinician, in turn, prepares for the visit and saves time by reviewing them before seeing the patient [116], then, scans the systems review and records the number of positives on the symptom checklist and reviews the recent medical history in order to improve accuracy and completeness of critical information [20, 70, 93, 98, 106].

However, most visits of patients with RA to rheumatologists include neither a formal quantitative joint count nor use of questionnaires [68]. This situation may be due to limitations that PROs instruments have, which includes the fact that about 20% of the patients may need some help to complete even a simple self-report questionnaire [117]. Furthermore, floor effects are seen, that is, patients may have normal HAQ scores but nonetheless feel that there are functional limitations [104]. Other times, the physicians do not check the patient's clinical status, and the patients felt unhappy after completing questionnaires if there was no evidence that the information was reviewed by a health professional [104]. Some authors have reported that specialized questionnaires are too cumbersome for usual clinical care, and short questionnaires are needed. 

Sometimes the PROs instruments are nonspecific and measurements may show improvement in the patient status due to other situations unrelated to RA. They are subject to cultural differences (i.e., pain scores are highest in Latin Americans patients and lowest in Asian patients), must be translated into and validated in various languages, and may be subject to gaming by certain patients to give desired answers [20, 78, 110].

Other authors had shown disparities between physician and patient measurements. Studenic et al. [118] found patients and physicians often differed in theperceptionofRAdiseaseactivity, quantified by VAS-Global and MD-Global. This was due to a worseperception of pain by the patient, while for SJC, the worse perception was by the physician. The two discrepancies explain 65% of the discordance between patient and physician measurements. 

### 4.4. Limitations and Conclusions

The present study had some limitations. The focus groups could be one of them since some patients may influence others and affect their answers. This could raise questions about its reproducibility both collectively and individually. In addition, measurements of test–retest reliability were not done because each focus group gathered only once, and an intragroup correlation cannot be done. 

Through this study, we can conclude that PROs can be administered collectively without any specialized guidelines thus providing a space for group education. Therefore, PROs can be done in rheumatology practice using the processes and instruments described above. This practice will help to advance rheumatology as a specialty and improve the lives of millions of people with RA due to the fact that patient questionnaires can be collected easily, completed in a limited time, and done in all clinical practices. These questionnaires can be completed for patients at each visit regardless of gender, educational level, age, or duration of disease as demonstrated here. PROs are not intended to be a substitute for objective scores such as the DAS28 determined during physician visits, in other words, they do not replace the clinical judgment or a careful articular examination. On the contrary, they are complementary. Together, they act synergistically and allow the physician and patient to reach a consensus evaluation in order to achieve and support a long-term improvement of the patient's condition through better treatment. 

We encourage clinicians to implement quantitative measurements about patient status in RA using PROs, since they are standardized, efficient, and effective. These appear wellsuited to a continuous quality improvement approach in standard patient care, contributing to provide data regarding functional status, pain, global status, fatigue, and psychological status that cannot be obtained any other way. We hope that implementation in rheumatology centers could provide the benefits described in this paper, increasing treatment adhesion, costs reduction and lead to a better outcome in RA.

## Figures and Tables

**Table 1:** Characteristics of 135 patients with RA evaluated in the current study.

Characteristic	Mean ± SD
Age	53.63 ± 11.28
Age at onset	40.5 ± 12.14

Characteristic	Median ± IQR

Duration of the disease	12 ± 14
Educational level (years)	11 ± 10
Body mass index	24.14 ± 5.69
Waist-hip ratio	0.92 ± 0.09
Systolic blood pressure	120 ± 20
Diastolic blood pressure	70 ± 11
C-Reactive protein	0.39 ± 1.06
DAS28	2.75 ± 1.30
HAQ	0.99 ± 1.19
TJC physician	2 ± 4
SJC physician	2 ± 4
TJC patient	7 ± 13
SJC patient	4 ± 8
SDAI	13.72 ± 14.45
CDAI	13 ± 13.50

Variable	*n*/*N* (%)

Sociodemographic	

Female	106/135 (78.5)
Low educational level	59/133 (44.4)
Low socioeconomic status	52/132 (39.4)
Ever smoking	50/134 (37.3)
Household duties	49/135 (36.3)

Clinical aspects	

Diabetes	5/135 (3.7)
Dyslipidemia	28/135 (20.7)
Hypertension	56/135 (41.5)
Thrombosis	6/135 (4.4)
Osteoporosis	42/135 (31.1)
Occlusive arterial disease	3/135 (2.2)
Cardiovascular disease	63/135 (46.7)
Abnormal body mass index	61/133 (45.9)
Abdominal obesity	106/134 (79.1)
Physical activity	44/135 (32.6)

RA characteristics	

Typical morning stiffness	100/134 (74.6)
Duration disease > 10 years	78/135 (57.9)
Erosions	71/108 (65.7)
Nodules	40/135 (29.6)
EAMs	47/135 (34.8)
EAMs with CVD	87/135 (64.4)
Rheumatoid factor +	106/124 (85.5)
Anti CCP +	58/70 (89.2)
Methotrexate	121/135 (89.6)
DMARD	128/135 (42.2)
Antimalarials	106/135 (78.5)
Steroids	122/135 (90.4)
Biological agents	57/135 (42.2)
Alternative medicine	73/130 (56.2)

Autoimmunity	

Systemic lupus erythematosus	1/135 (0.7)
Autoimmune thyroid disease	13/135 (9.6)
Sjögren's syndrome	4/135 (3)
Antiphospholipid syndrome	2/135 (1.5)
Vitiligo	1/135 (0.7)
Scleroderma	1/135 (0.7)
Polyautoimmunity	19/135 (14.1)
MAS	3/135 (2.2)
Familial autoimmunity FDR	22/135 (16.3)
Familial autoimmunity SDR	5/135 (4.4)
ANAs +	63/99 (63.6)

RA: rheumatoid arthritis; SD: standard deviation; IQR: interquartile range; DAS28: disease activity score; HAQ: health assessment questionnaire; TJC: tender joint count; SJC: swollen joint count; SDAI: simplified disease activity index; CDAI: clinical disease activity index; EAMs: extraarticular manifestations; CVD: cardiovascular disease; Anti-CCP: anticyclic citrullinated peptide; DMARD: disease modifying-antirheumatic drugs; MAS: multiple autoimmune syndrome; FDR: first degree relatives; SDR: secondary-degree relatives; ANAs: antinuclear antibodies.

**Table 2:** Agreement and correlations* between values finding by PROs and physician**.

Values physician/ Values patient	SJC physician	TJC physician	DAS28	MD-Global	CDAI	SDAI
SJC patient	0.772^b^	0.499	0.525	0.531	0.563	0.541
TJC patient	0.429	0.75^b^	0.552	0.493	0.611	0.598
RADAI	0.393	0.604	0.56	0.399^a^	0.667	0.646
RAPID3	0.372	0.594	0.523	0.361^a^	0.731	0.706
RAPID4	0.402	0.625	0.562	0.395^a^	0.75	0.726
RAPID5	0.53	0.709	0.662	0.511^a^	0.829	0.851
MDHAQ	0.246^d^	0.491	0.442	0.304^a^	0.531	0.531
VAS-Global	0.396	0.583	0.517	0.026^c.e^	0.754	0.725
VAS-Pain	0.323	0.508	0.434	0.314^a^	0.632	0.606

*Correlations were evaluated by spearman's rank correlation coefficient. except:

^a^Correlation by Kendall's Tau *b* test.

^b^Agreement by Kendall's *W* test.

^c^Agreement by Weighted kappa.

**All data *P* < 0.0001, except in ^d^ *P* = 0.004 and ^e^ *P* = 0.241.

PROs: patient-reported outcomes; SJC: swollen joint count; TJC: tender joint count; DAS28: disease activity score with 28 joints; MD-Global: global assessment by visual scale analogue from physician; CDAI: clinical disease activity index; SDAI: simplified disease activity Index; RADAI: self-administered rheumatoid arthritis disease activity index; RAPID: routine assessment of patient index data; MDHAQ: multidimensional health assessment questionnaire; VAS-Pain: pain assessment by visual scale analogue; VAS-Global: global evaluated by patient in visual scale analogue.

**Table 3:** Predictors of DAS28 with PROs variables.

	β*	*P*
Constant	2.021	<0.001
RAPID4	0.037	0.03
Patient SJC	0.042	<0.001
RADAI	0.073	0.48
Patient TJC	0.009	0.49
Gender	−0.123	0.44
RA duration	0.029	0.83
Education level	0.127	0.35

PROs: patient reported outcomes; *β*: beta coefficient; *P*: *P* value; RAPID: routine assessment of patient index data; SJC: swollen joint count; TJC: tender joint count; RA: rheumatoid arthritis.

*The beta coefficients give a measure of the contribution of each variable to the model. A large value indicates that a unit change in this predictor variable has a large effect on the criterion variable (DAS28).

## Abbreviations

AD: Autoimmune disease
AITD: Autoimmune thyroid disease
ANAs: Antinuclear antibodies
Anti-CCP: Anticyclic citrullinated peptid
APS: Antiphospholipid syndrome
BMI: Body mass index
CDAI: Clinical disease activity index
CRP: C-reactive protein
DAS28: Disease activity score-28 joints
DMARDs: Disease modifying antirheumatic drugs
EAMs: Extraarticular manifestations
ESR: Erythrocyte sedimentation rate
FDR: First-degree relatives
HAQ: Health assessment questionnaire
IQR: Interquartile range
MAS: Multiple autoimmune syndrome
MD-Global: Global assessment by visual scale analogue by physician
MDHAQ: Multi-dimensional health assessment questionnaire
PROs: Patient-reported outcomes
RA: Rheumatoid arthritis
RADAI: Rheumatoid arthritis disease activity index
RAPID: Routine assessment of patient index data
RF: Rheumatoid factor
SAI: Selfadministered index
SD: Standard deviation
SDAI: Simplified disease activity index
SES: Socioeconomic status
SJC: Swollen joint count
SLE: Systemic lupus erythematosus
SS: Sjögren's syndrome
SSc: Scleroderma
TgAb: Antithyroglobulin protein
TJC: Tender joint count
TSH: Thyrotropin
TPOAb: Anti-thyroperoxidase enzyme
VAS-Global: Global evaluated by patient in visual scale analogue
VAS-Pain: Pain visual scale analogue
WHR: Waist-to-hip ratio.
